# Changes in Empathy and Mental Resilience in Health Professionals After Completing the Certified “Generic Instructor Course” Seminar

**DOI:** 10.7759/cureus.64752

**Published:** 2024-07-17

**Authors:** Styliani Paliatsiou, Theodoros Xanthos, Vasiliki Efthymiou, Ioannis Zervas, Paraskevi Volaki, Rozeta Sokou, Nicoletta Iacovidou

**Affiliations:** 1 Second Department of Obstetrics and Gynecology, Aretaieio Hospital, National and Kapodistrian University of Athens, Athens, GRC; 2 Department of Cardiology, School of Health Sciences, University of West Attica, Athens, GRC; 3 Department of Biostatistics, University Research Institute of Maternal and Child Health and Precision Medicine, National and Kapodistrian University of Athens, Athens, GRC; 4 First Department of Psychiatry, Eginition Hospital, National and Kapodistrian University of Athens, Athens, GRC; 5 Department of Neonatal, Aretaieio Hospital, National and Kapodistrian University of Athens, Athens, GRC; 6 Department of Pediatrics, Neonatal intensive Care Unit, General Hospital of Nikaia-Piraeus "Agios Panteleimon", Athens, GRC

**Keywords:** simulation training, skills, interpersonal, generic instructor course, empathy

## Abstract

Introduction: Factors that may affect the performance of healthcare professionals performing resuscitation include stress, social profile, fatigue, empathy, and resilience. Interpersonal skills are required for better performance. This study aimed at evaluating the change in empathy and mental resilience in health professionals who have the status of instructor potential achieved after successfully completing a certified training/intervention course and want to develop/certify as course instructors.

Methods: Healthcare professionals attended the Generic Instructor Course (GIC), a two-day course training instructor candidates from different training courses. Empathy and the cultivation of mental resilience of adult healthcare professional trainers were measured in order to investigate whether participation in a simulated training process can influence these characteristics of the trainer and how these characteristics interact with the training process. Four measurements were recorded: (i) baseline (before the GIC course), (ii) after the course, (iii) follow-up after one month, and (iv) follow-up after three months.

Results: Ninety participants in the GIC course were the study sample. Participants showed statistically higher empathy after participation in the GIC vs. baseline, one-month, and three-month follow-up (p = 0.023). Resilience did not reveal any statistical difference, after the participation in the GIC and follow-up measurements (p = 0.084). For both variables (empathy and resilience), demographics did not have any association with the variables.

Conclusions: Besides its primary aim of training and certifying future instructors in resuscitation courses, the GIC also had a positive impact on the participants' empathy.

## Introduction

A review study of 258 cases involving malpractice claims reported that 41% were due to inexperience/lack of technical competence, while 24% were due to poor communication between healthcare professionals [1]. Similar studies suggested that investing in training and improving the capacity of employees was considered satisfactory, taking into consideration that adequate and well-staffed health services must be a priority [2]. However, in real life investing time in training on leadership and communication skill development remains very low. Therefore, "leadership gaps" are identified, i.e. gaps in the need to develop leadership and management skills at all levels, and therefore the possibility of training everyone (i.e. employees, businesses, and job seekers) is considered increasingly necessary [3].

Scholars define the relevant skills as the triad of interpersonal relationships, e.g. communication and teamwork; cognitive skills, e.g. decision-making and situational awareness; and personal cognitive skills e.g. coping with stress, cultivating mental resilience, coping with fatigue with empathy, and projecting emotional intelligence. The American Association of Critical Care Nurses claims that communication skills or interpersonal skills of healthcare professionals are an integral part of healthcare provision, as they contribute to safe patient care and to healthy work atmosphere, where it is observed that personal qualities, characteristics, and behaviors interact and influence the type and timing of the healthcare product delivered [4]. Research reflects the interest in challenging the development of interpersonal skills that can impact both employee performance and health, and consequently patient management. Various approaches were proposed for the design of educational development - experiential learning programs, which contribute to the preparation of health professionals by combining updated theoretical and clinical expertise with the expression of an optimized level of interpersonal development at work [5]. In the health sector, leadership, responsibility, empathy, decision-making, communicating and managing in an effective and effective manner as well as emotional intelligence (interpersonal skills) receive particular attention in the current literature. Recent studies have indicated that factors that can affect the performance of healthcare professionals performing resuscitation include stress, social profile, fatigue, empathy, and resilience [6].

Empathy is someone’s ability to empathize, understand the situation and behavior of the other person, and share emotions and thoughts with emotional involvement. It consists of a triptych of concept dimensions: (i) the cognitive dimension, which is related to understanding the feelings of the other person; (ii) the affective dimension, which concerns the emotional form; and (iii) the communicative dimension, which is related to the expression-transmission of verbal messages through an egalitarian counseling relationship [7]. It is categorized into (a) cognitive, i.e. the ability of the therapist to put himself in the position of the person being treated and perceive the way the person thinks and reacts, and (b) thymic, which concerns the ability of the therapist to be able to perceive the feelings of the person being treated [8]. For health professionals involved in therapeutic practices, the ability to communicate is a key tool for achieving the goal. Studies confirmed that poor communication with patients and inability to empathize lead to failure to comply with treatment and to unsatisfactory outcomes of the applied treatment to the patient [9]. It also appears that simulation training improves empathy and general communication skills more than simple teaching of trainee health professionals [10]. Research data specific to those who use any form of resuscitation is limited.

Mental resilience is someone’s ability to react positively and adapt to adverse events [11]. It includes a set of characteristics that interact with each other dynamically in order to return the individual to his/her previous state, successfully coping with difficulties. For healthcare professionals, the term refers to an individual's ability to perceive the complexity of problems, manage stressful situations, and minimize mental and physical stress [12]. Especially for health professionals who apply for mindfulness-based programs, this characteristic is of great importance, as studies have shown that the inability to cope with stressful conditions affects the quality of life of health professionals [13].

The purpose of this study is to evaluate the change in specific characteristics such as empathy and mental resilience in health professionals who have the status of instructor potential achieved after successfully completing a certified training/intervention course and want to develop/certify as course instructors.

## Materials and methods

Sample

Ninety healthcare care professionals who participated in the Generic Instructor Course (GIC), having successfully completed Advanced Life Support (ALS), Immediate Life Support (ILS), European Trauma Course (ETC), Newborn Life Support (NLS), and others organized by the Hellenic Society for Cardiopulmonary Resuscitation (HSCR) were the study subjects. The sample was selected using cluster sampling.

Procedure

Current research is designed as an interventional uncontrolled study. The training course/intervention under study is the GIC, organized and certified by the European Resuscitation Council (ERC), and HSCR. The aim of the course is to provide the necessary training framework to prepare future instructors for the delivery of resuscitation courses, strictly in the context of the training process and not in resuscitation per se. As a prerequisite, participants must hold a valid instructor potential status from training courses such as ALS, ILS, ETC, and NLS.

The GIC is conducted in various centers in Europe, including Greece. The course is approved by the ERC and has a specific form structure, homogeneity in content, teaching strategy, and formative assessment, and it is standardized irrespective of the center where it is conducted. The GIC was created to address teaching skills that can be applied to all the following; (i) how to teach and facilitate learning within a small group discussion; (ii) how to teach and facilitate the learning of skills through objective assessment; (iii) how to conduct a teaching module on a specific topic, depending on the course, e.g. simulated cardiac arrest; (iv) how to conduct constructive and corrective teaching to promote further improvement of knowledge; (v) how to conduct a simulation examination without interference, respecting the examination code and objectivity e.g. cardiac arrest, for final assessment.

The GIC is a two-day course (of 20 hours duration). Participants are divided into four groups. An Educator, a Course Director, and eight Instructor Trainers train future instructors. The GIC program is depicted in detail in Table 1. At the end of the first day, in the session “The role of the Coach; The Coaching session”, the educator discusses what coaching is; Coaching (rationale for ERC courses), key aspects of effective coaching - peer support and feedback, discussion on strategies for 1:1 coaching, how it is this used on provider courses (expectations and problems). After this, a discussion is led by the educator based on a video of a simulation performance or other method defined by the educator.

**Table 1:** Generic Instructor Course (GIC) schedule

Time	Learning outcomes and topics to be taught
Day 1	
08.00-09.00	Faculty Meeting/Registration
09.00-09.20	Introduction and Welcome
09.20-10.00	Get to Know the Equipment Introduction in Groups
10.00-10.15	Refreshment Break
10.15-10.35	Adult Learning and Effective Teaching 1
10.35-10.55	Skills Teaching and Continuous Assessment, Discussion
10.55-12.25	Skills Teaching With Continuous Assessment Practice 1; Allocation of Groups Into Rooms
12.25-13.10	Lunch Break (45’)
13.10-13.30	Adult Learning and Effective Teaching 2
13.30-13.50	Teaching Simulations and Debriefing Demonstration and Discussion: Demo Scenario Related to Candidates’ Backgrounds
13.50-14.05	The ERC Debriefing Strategy, Discussion
14.05-16.05	Simulation Teaching Practice 1 Allocation of Groups Into Rooms
16.05-16.20	Refreshment Break
16.20-16.35	Facilitating Small Group Teaching Demo Scenario Related to Candidates’ Background
16.35-17.35	Small Group Teaching Practice 1: Allocation of Groups Into Rooms
17.35-18.00	The Role of the Coach; the Coaching Session Allocation of Groups Into Rooms
18.00-18.30	Faculty Meeting
Day 2	
08.00-08.15	Coaching Time With Tea/Coffee
08.15-08.30	Adult Learning and Effective Teaching 3
08.30-09.00	NTS - Plenary Interactive Discussion Fishbowl Discussion on NTS or Discussion Based on a Video of a Simulation Performance or Other Method Defined by the Educator
09.00-09.45	Principles of Summative Assessment in Simulation Plenary and Demo Scenario Related to Candidates’ Background
09.45-10.00	Refreshment Break
10.00-12.00	Simulation Assessment, Practice Allocation of Groups Into Rooms
12:00-12:45	Lunch Break
12.45-13.45	Skills Teaching With Continuous Assessment Practice 2; Allocation of Groups Into Rooms
13.45-14.30	Small Group Teaching Practice 2 Allocation of Groups Into Rooms
14.30-14.45	Refreshment Break
14.45-16.15	Simulation Teaching Practice 2 Focus = NTS Debriefing Allocation of Groups Into Rooms
16.15-16.30	The Role of the Instructor, Discussion
16.30-16.45	Faculty Meeting, Review of GIC-Candidates
16.45-16.55	Course Closure
16.55-17.15	Faculty Meeting

The training of empathy and the cultivation of mental resilience of adult health professional trainers (part of interpersonal skills) is a priority, as it will be shown whether participation in such a program of the simulated training process - experiential learning can change or influence these characteristics of the participants and how these characteristics change or interact due to the training process and whether this change remains after a period of time. Therefore, four measurements were recorded: (i) baseline (before the GIC, (ii) after the course/certification, (iii) follow-up after one month, (iv) follow-up after three months.

Tools

Participation in the study was on a volunteer basis and informed consent was signed prior to recruitment. The demographics of the participants were recorded.

To measure empathy, the Toronto Empathy Questionnaire (TEQ) was used. It consists of 16 items that are scored on a 5-point Likert scale (0 = Never, 1 = Rarely, 2 = Sometimes, 3 = Often, 4 = Always), in order to measure the degree of empathy. The score is derived from the sum of the responses and ranges from 0 to 40, with higher scores being indicative of a greater degree of empathy. The questionnaire was validated in Greek and demonstrated high internal consistency and reliability (Cronbach's alpha=0.72) [14].

Mental resilience was measured with a short tool, the Connor and Davidson Resilience Scale (CD-RISC), which was translated and validated in the Greek population [15]. It consists of 25 items that are scored on a 5-point Likert scale, with a total score ranging from 0 to 100. Higher scores are indicative of greater mental resilience. The CD-RISC showed excellent internal consistency in the Greek population (Cronbach’s alpha = 0.925), as well as excellent test-retest reliability (intra-class correlation coefficient = 0.925) [15].

Statistical analysis

Statistical analysis of the study was conducted using the statistical software IBM SPSS (IBM Statistical Package for Social Sciences for Windows, Version 25.0. Armonk, NY: IBM Corp). Values were expressed as mean, standard deviation, absolute, and relative frequencies. In order to examine the effect of GIC at four different time points, repeated measures of analysis of variance (ANOVA) were used. Post-hoc least significant difference analysis was used to investigate the time point with different levels of empathy or resilience. A mixed model of repeated measures of ANOVA was used to examine the interaction of several demographics on the effect of GIC at four different time points. Dealing with missing data, while the assumption of missing data completely at random was satisfied, the listwise deletion was applied as the most frequently used method in handling missing data in statistical software packages such as SPSS. The statistically significant level was set at 0.05.

## Results

Demographic and other characteristics

The demographics of the participants are shown in Table 2. The GIC was the participants’ first training program, and 69.0% answered that they did not have any prior experience in courses related to education. For 46.7%, the incentive for certification was the improvement of their curriculum vitae, for 13.3% was work needs, for 15.6% was curriculum vitae and work needs and finally for 16.7% was a personal improvement.

**Table 2:** Demographic and other characteristics of the sample (N=90) Values are referred to as absolute and relative frequencies (%).

Characteristics		n	%
Gender	Male	31	34.4
	Female	59	65.6
Age (years)	24-30	57	63.3
	31-35	14	15.6
	36-40	2	2.2
	40-45	12	13.3
	46-50	4	4.4
	51+	1	1.1
Residency	Other province	31	34.8
	Attica	58	65.2
Family status	Single	63	70.0
	Married	16	17.8
	Living together	11	12.2
Educational level	Secondary education	1	1.1
	Technological	3	3.4
	University	45	51.1
	Master	30	34.1
	PhD	6	6.8
	Post-Doc	3	3.4
Employment	Unemployed	5	5.6
	Self-employed	7	7.8
	Public sector	72	80.0
	Private company	6	6.7
Work-study relevance	Yes	85	98.8
	No	1	1.2
Employment status	Permanent	23	28.7
	Contract holder	57	71.3
Work experience (years)	Less than 5 years	60	69.8
	6-10	16	18.6
	11-15	5	5.8
	16-20	2	2.3
	21+	3	3.5
Prior experience in training process	Yes	27	31.0
	No	60	69.0
Incentive for certification for an adult trainer	Curriculum vitae	42	46.7
	Work needs	12	13.3
	Other	7	7.8
	Curriculum vitae and work needs	14	15.6
	Personal improvement	15	16.7

Levels of empathy and resilience

Healthcare professionals were asked to complete two questionnaires, as mentioned in the method section (Table 3). The TEQ total score was statistically different in four time points. More specifically, post-hoc analysis showed differences between after the GIC (M ± SD = 52.00 ± 6.35) vs. baseline (M ± SD = 50.78 ± 5.03), one-month (M ± SD = 50.81 ± 5.67) and three-month (M ± SD = 50.32 ± 6.64) follow-up measurements. For the CD-RISC total score and its subscales, non-significant differences were revealed between different time points (p = 0.08 for the total score).

**Table 3:** Levels of empathy and resilience at different time points GIC: Generic Instructor Course; TEQ: the Toronto Empathy Questionnaire; CD-RISC: the Connor-Davidson Resilience Scale Values are referred to as mean and standard deviation (SD). P-value was calculated using analysis of variance (ANOVA) for repeated measures. * Post-hoc least significant difference analysis showed differences between after the GIC time point vs. baseline, one-month, and three-month follow-up measurements.

Scale	Baseline	After the GIC	One-month follow-up	Three-month follow-up	p-value
TEQ total score	50.78 ± 5.03	52.00 ± 6.35	50.81 ± 5.67	50.32 ± 6.64	0.02*
CD-RISC total score	67.06 ± 9.29	69.03 ± 9.06	69.21 ± 11.32	68.82 ± 12.37	0.08
Notion of personal competence, high standards, and tenacity	22.64 ± 3.66	23.58 ± 3.45	23.26 ± 4.21	23.22 ± 4.64	0.10
Trust in one’s instincts, tolerance of negative affect, and strengthening effects of stress	17.81 ± 3.18	18.14 ± 3.33	18.56 ± 3.75	18.32 ± 3.92	0.18
Positive acceptance of change, and secure relationships	15.01 ± 3.71	15.08 ± 2.20	15.26 ± 2.36	15.31 ± 2.43	0.67
Control	8.18 ± 1.75	8.61 ± 1.80	8.39 ± 1.89	8.39 ± 1.92	0.15
Spiritual influences	3.41 ± 1.94	3.62 ± 2.00	3.76 ± 2.17	3.58 ± 2.11	0.10

The examination of the interaction of several demographic characteristics such as gender, age, and educational level is presented in Table 4. Non-significant differences were revealed for all characteristics in both empathy and resilience (p > 0.05).

**Table 4:** Levels of empathy and resilience at different time points according to different demographic characteristics GIC: Generic Instructor Course; TEQ: the Toronto Empathy Questionnaire; CD-RISC: the Connor-Davidson Resilience Scale Values are referred to as mean and standard deviation (SD). P-value was calculated using analysis of variance (ANOVA) for repeated measures (mixed model).

Scale	Baseline	After the GIC	One-month follow-up	Three-month follow-up	p-value
TEQ total score					
Gender					0.50
Male	47.74 ± 4.48	49.52 ± 6.02	48.13 ± 5.75	46.77 ± 6.31	
Female	52.37 ± 4.57	53.31 ± 6.17	52.22 ± 5.13	52.19 ± 6.07	
Age					0.46
24-30 years	50.44 ± 4.76	51.81 ± 4.65	50.12 ± 5.24	50.26 ± 6.58	
31+ years	51.36 ± 5.48	52.33 ± 8.60	52.00 ± 6.25	50.42 ± 6.84	
Educational level					0.39
Bachelor	50.15 ± 4.00	51.98 ± 5.04	50.08 ± 5.43	49.85 ± 6.23	
Post-graduate studies	51.41 ± 6.16	51.59 ± 7.70	51.38 ± 5.99	50.38 ± 7.12	
CD-RISC total score					
Gender					0.64
Male	68.16 ± 10.55	68.61 ± 10.18	69.35 ± 11.33	68.71 ± 12.65	
Female	66.47 ± 8.59	69.25 ± 8.49	69.14 ± 11.41	68.88 ± 12.33	
Age					0.67
24-30 years	66.46 ± 8.89	68.14 ± 8.71	67.88 ± 11.13	67.53 ± 11.41	
31+ years	68.09 ± 10.00	70.58 ± 9.57	71.52 ± 11.45	1.06 ± 13.79	
Educational level					0.64
Bachelor	66.08 ± 9.74	67.96 ± 9.63	67.79 ± 11.63	66.65 ± 11.32	
Post-graduate studies	67.87 ± 8.85	70.21 ± 8.46	70.56 ± 11.00	70.72 ± 13.14	

## Discussion

The GIC was developed and updated by the Advanced Life Support Group and the Resuscitation Council of the United Kingdom [16]. The GIC’s purpose is the development of capable instructors, and it contributes to a combination of a learning experience giving to instructor candidates the required skills, knowledge, and mental state to provide effective training [16].

The definition of skills could be described as the activation of a combination of knowledge and mindset in a particular situation towards accomplishing specific outcomes [17]. The interpersonal skills could be defined as a set of methodological and practical knowledge that is dynamically activated and manifested in performance [18]. The contribution of interpersonal skills was proven in individual health, success, well-being, and collective progress.

Interpersonal skills contribute to performance in the workplace as well as in training programs and particularly in self-efficacy and adaptive performance [19]. Although the development of these skills is crucial in order to foster employability [20], there are questions about how possible is to train these skills [21] and about the cost of the time of training interpersonal skills [19]. In contrast with professional skills that can be achieved in an educational institution, interpersonal skills training often requires long-term interventions, and experiential methods of training, i.e. mentoring [22]. A recent study revealed the association between interpersonal skills and performance by studying how interpersonal skills metacognition are related to self-efficacy and adaptive performance in a transformational context [19]. Adaptive performance has a strong association with coping with an affection for resilience and adaptation when faced with innovation [23]. Another study reported that the optimum model of a contemporary educator should obtain skills reflective of a real environment, so these skills development (such as effectiveness, perseverance, stress resilience, and mental and physical health) could be used in the improvement of lifelong learning and adult education [24].

Empathy as a factor of interpersonal skills plays an important role in healthcare professionals, as it is related to decreased levels of burnout and higher satisfaction in the workplace [25,26]. In a similar study with an intervention with a combination of learning methods, such as lectures, simulations with trained actors, group debriefings, and a photo language session called “Program to Enhance Relational and Communication Skills for Intensive Care Unit”, physicians showed higher levels in empathy, preparation, communication skills, relational skills, confidence, emotion­al awareness, and self-reflection [27]. This outcome was confirmed in the present study, while healthcare professionals showed higher levels of empathy after the GIC. Nevertheless, this improvement did not remain at the same levels in the follow-up measurements. Other teaching learning strategies, such as psychosocial role-playing skills, have been proven to enhance nurses' develop interpersonal skills and nurses' healthcare interactions combined with communication, interpersonal interaction, empathy, active listening, teamwork, delegation, and/or professionalism [28].

Resilience as a factor of interpersonal skills was established in various studies aimed at the improvement of different personal and social capabilities frameworks after adapted interventions. Outcomes after a learning intervention offered to medical students showed that skill sets were significantly correlated to the annual academic performance of the students in year 1, but were not associated with their annual academic performance in year 2 [29]. Analysis performed after the digital training platform and aiming at increasing physicians’ interpersonal communication skills after the COVID-19 pandemic revealed that healthcare professionals require skills that exceed the cultivation of resilience and flexibility [30]. Similar results confirmed the GIC as an intervention without validation of the outcomes with statistical significance. Despite the non-significant results, a tendency for higher levels of resilience was observed after the GIC, except for the dimension of positive acceptance of change and secure relationships.

All outcomes that were obtained after the GIC intervention are not associated with demographic characteristics such as gender, age, and educational level. This result suggests that the intervention affected all participants horizontally with the same impact.

Despite the reliability and validity of the study, the authors identified several study limitations that need to be acknowledged. All measures were collected using self-reported tools, so they did not ac­tual measure changes in behavior. Moreover, the generalization of the outcomes should be under consideration be­cause due to the nature of the seminar, as the control group was not involved.

## Conclusions

The GIC despite the obvious aim to train and certify future instructors in the organization of resuscitation courses also had an impact on participants’ empathy. Results, revealed after the GIC intervention, are not associated with demographic characteristics such as gender, age, and educational level. This outcome implies that the intervention affected the sample horizontally with the same impact. Although an improvement in resilience was also observed, this was not significant so it must be further investigated.
